# A Solitary Pulmonary Nodule: Pulmonary Amyloidosis

**DOI:** 10.5334/jbsr.1463

**Published:** 2018-01-31

**Authors:** Chloë Standaert, Vincent Herpels, Patrick Seynaeve

**Affiliations:** 1 University Hospital Ghent, BE; 2 AZ Groeninge Kortrijk, BE

**Keywords:** Amyloidosis, ^18^F-FDG PET-CT, Pulmonary nodule

## Clinical History

**Figure 1:**
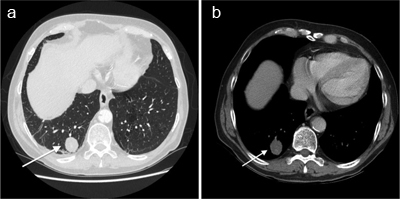
Contrast-enhanced CT scan of the abdomen shows a well-defined pulmonary nodule (27mm × 23 mm) in the right lower lobe.

A 67-year-old male and former smoker was admitted to the hospital because of abdominal pain. An abdominal CT scan revealed not only acute biliary pancreatitis, but also a pulmonary nodule in the right lower lobe (Figure 1).

**Figure 2:**
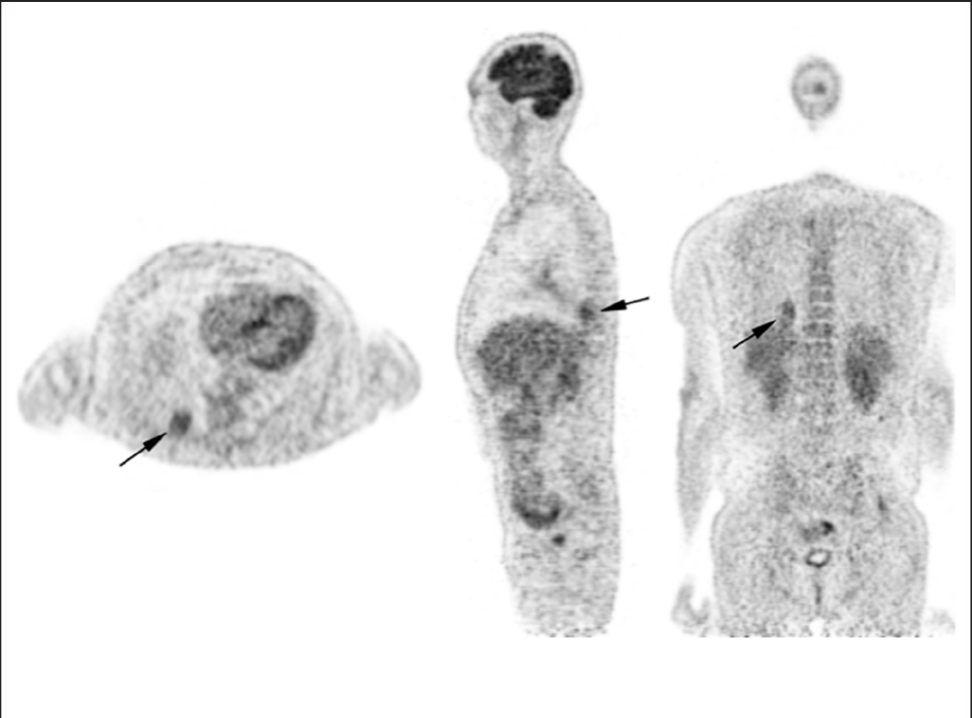
^18^F-FDG-PET shows a pulmonary nodule in the right lower lobe with high ^18^F-FDG uptake. There are no ^18^F-FDG-avid adenopathies.

On 18-Fluoro-deoxyglucose (^18^F-FDG) PET-CT scan the solitary pulmonary nodule posterior in the right lower lobe revealed a high ^18^F-FDG-uptake (Figure 2). No enlarged or high uptake lymph nodes nor distant metastasis were seen. So far, a malignant tumor was suspected. By CT-guided biopsy of the nodule, a specimen was obtained and revealed necrotic tissue without evidence of malignancy. Mediastinoscopy for mediastinal mapping was performed. All biopsies showed normal lymph node tissue and no malignancy.

Finally, a thoracoscopic right bilobectomy was performed. Histopathology of the specimens revealed an amyloid tumor with maximal diameter of 35 mm. Extensive diagnostic work-up for systemic amyloidosis remained negative. The diagnosis of pulmonary amyloidosis was confirmed.

## Comment

Amyloidosis refers to a variety of disorders associated with extracellular deposition of protein, caused by the aggregation of misfolded proteins. Localized amyloidosis refers to amyloid deposition in a single organ, usually amyloid light chain type, while in systemic amyloidosis concurrent involvement in other organs is described. Most cases of pulmonary amyloidosis are part of systemic amyloidosis and the diagnosis of localized pulmonary amyloidosis requires the absence of systemic amyloidosis.

Three main forms and CT patterns of pulmonary amyloidosis have been described: tracheobronchial amyloidosis, diffuse interstitial amyloidosis and nodular pulmonary amyloidosis [1]. Tracheobronchial amyloidosis is characterized by mural nodules and calcification of the thickened tracheobronchial wall, localized or multifocal. Patients present with stridor, dyspnea, recurrent pneumonia, or atelectasis. The differential diagnosis includes diffuse tracheal diseases such as relapsing polychondritis, granulomatosis with polyangitis, sarcoidosis, and inflammatory bowel disease.

Diffuse interstitial amyloidosis is the least common form and has the worst prognosis. It is characterized by ground-glass infiltrates, reticular opacities, interlobular septal thickening, nodules of 2–4 mm in size and consolidation in the subpleural region. It should be differentiated from other interstitial lung diseases, such as lymphangitic carcinomatosis.

In nodular pulmonary amyloidosis, single or multiple nodules appear radiologically with sharp and lobulated margins. Amyloid nodules in the lung parenchyma are usually incidental findings on chest radiographs. They are more often found in the lower lobes, in the subpleural or peripheral regions and should be considered in the differential diagnosis of pulmonary primary or metastatic neoplasms. Over time, the nodules may grow and cavitate, calcify, or resolve spontaneously.

^18^F-FDG PET-CT is emerging as a tool for the diagnostic work-up of pulmonary nodules to reduce inappropiate invasive diagnostic examination. However, ^18^F-FDG has little uptake in malignancies with low metabolic activity, such as bronchoalveolar cancer, carcinoid tumor, and mucinous adenocarcinoma. In addition, certain non-malignant conditions such as tuberculosis, sarcoidosis, and rheumatoid nodules demonstrate high metabolic rates. Our case and some other rare reported cases showed lung nodules of pulmonary amyloidosis with moderate ^18^F-FDG uptake. Therefore, positive results of ^18^F-FDG PET-CT should be interpreted with caution in differentiating pulmonary amyloidosis from malignant lesions. The final diagnosis of localized pulmonary amyloidosis requires histological confirmation. CT-guided fine needle aspiration biopsy may avoid unnecessary invasive surgical resection.

Prognosis and treatment depend on the type and distribution of the disease. Localized forms can usually be managed conservatively, whereas systemic forms are treated with chemotherapy (primary amyloidosis) and anti-inflammatory agents.
